# Morphometric Analysis of Achilles Tendon Structure and Its Significance: A Cadaveric Study

**DOI:** 10.7759/cureus.32172

**Published:** 2022-12-03

**Authors:** Yuvaraj Maria Francis, Sameen Taj, Sankara Narayanan G, Balaji Karunakaran, Kirthika CP, Sankaran PK, Akhilesh Ravichandran, Gunapriya Raghunath, Sarah Senthilkumar, Zareena Begum

**Affiliations:** 1 Anatomy, Saveetha Institute of Medical and Technical Sciences, Chennai, IND; 2 Anatomy, Saveetha Medical College and Hospital, Chennai, IND; 3 Anatomy, Saveetha Medical College, Saveetha Institute of Medical and Technical Sciences, Chennai, IND; 4 Anatomy, Sri Ramachandra Institute of Higher Education and Research, Chennai, IND; 5 Anatomy, All India Institute of Medical Sciences (AIIMS) Mangalagiri, Vijayawada, IND; 6 Pathology, Saveetha Medical College and Hospital, Chennai, IND; 7 Anatomy, All India Institute of Medical Sciences, Mangalagiri, Mangalagiri, IND; 8 Anatomy, Saveetha Medical College, Chennai, IND

**Keywords:** and tedinopathy, morphometry, histology, cadaver, achilles tendon

## Abstract

Introduction: Achilles tendon is crucial for gait, and chronic Achilles tendinopathy can have a substantial impact on an individual’s work and active involvement in physical or sports activity, and overall quality of life.

Objectives: This research was to determine the macroscopic and microscopic anatomy of Achilles tendons in cadavers.

Materials and methods: This experimental study was conducted in the Department of Anatomy, Saveetha Medical College, Thandalam, from March to August 2022. A total of 60 formalin-perfused cadavers (38 males and 22 females) were dissected to study their morphometry (length, width, thickness) and histology. The data was tabulated in MS excel and analyzed statistically using unpaired ‘t-test and one-way ANOVA in SPSS Software 17.0 (IBM Corp., Armonk, NY).

Results: The mean length of the Achilles tendon was significantly higher in males than in females and similarly, the length on the right side was significantly higher than on the left side (p<0.005). The width and circumference were statistically higher in females than, males whereas, the histological features were similar in both males and females.

Conclusion: The better understanding of Achilles tendon morphometry in cadavers always aids in the diagnosis and surgical repair of tendinopathy, rupture, and degenerative change. The knowledge will be helpful for the surgeons during the repair and reconstruction of the injured tendon.

## Introduction

Tendons are made up of dense regular connective tissue that conveys force from muscular tissue to bones. The Achilles tendon (AT), the strongest tendon in the human body, is made up of regularly arranged dense connective tissue with type I collagen fibers, set as fascicles. The regularly arranged collagen fibers (type I) have enough tendency to withstand the tensile and compressive loads during activities of daily living (ADL) such as walking, running, and jumping [1-3]. The continual stress in collagen fibers alters the property, resulting in repetitive microtears, which may cause a decrease in mechanical strength. The tendocalcaneum or AT is formed by the confluence of gastrocnemius and soleus muscles which get attached to the posterior surface of the calcaneum and result in plantar flexion at the ankle joint [4]. These muscles are typically large and powerful through AT, they provide propulsive force in walking, running, and jumping. The length of AT ranges from 8 to 17 cm with 90º spiral orientation which is due to the medial rotation of the lower limb bud during the development [5-9]. The aging and overweight overloads the AT which alter the cellular and molecular components resulting in damage to AT. AT injury varies from mild to severe forms such as tendinopathy, tendinitis, and rupture [10-15]. Previously much research has been undertaken on AT were done using ultrasonography and MRI. While in cadavers, only minimal studies have been done. The aim of this study is to determine the morphometry, and histology of AT in both male and female cadavers of the south Indian population and to compare with the previous studies. The parameters of any structure in a cadaver differ to an extent from living individuals, although the cadaveric study remains a viable option to understand the macroscopic and microscopic structure. The literature review revealed that the study of the AT in laboratory animals, all of it gave less importance to the histology, hence this study was taken. The knowledge of morphometry will serve as an important landmark in the anthropometric evaluation and biomechanical characteristics. In addition, it will also be helpful for the orthopedic surgeons and rehabilitation team for novel approaches in surgical and non-surgical management.

## Materials and methods

One hundred twenty lower limb dissections of tendocalcaneum in the posterior compartment of legs were undertaken in a total of 60 cadavers (female: 22 and male: 38). This study was done in the Department of Anatomy, Saveetha Medical College and Hospital, Chennai. The study was preceded after obtaining proper institutional human ethical clearance (015/02/2022/IEC/SMCH). The cadavers with trauma or surgery in the lower limb were excluded. The corpses were placed in the prone position with slightly abducted and lateral rotated lower limbs to dissect the AT. The region was meticulously dissected to trace the AT from the point of formation to its site of insertion. Two pins were taken one was placed at the end of formation and another at the level of insertion. The morphometry of AT was obtained with the help of measuring scale, thread, and digital vernier caliper (Mituto yo digital vernier caliper with sensitivity to 0.1mm).

The following parameters were included for the study such as length of AT (from its formation to insertion), proximal width (PWAT) and circumference of the AT (near its formation) (PCAT), middle width (MWAT) and circumference of the AT (midway between formation and insertion) (MCAT), distal width (DWAT) and circumference of the AT (near its insertion) (DCAT).

The histology of the AT was done by procuring tissues from the specimens and the dehydration process was done by placing the tissues in the graded alcohol solutions, embedded in the paraffin sections followed by cutting of the sections at a thickness of 5µm with a rotatory microtome (INCO MRM-1120). The tissue slides were stained with hematoxylin and eosin (H and E) and observed under 10X magnifications with the light microscope the slides were then photographed with Image J software.

Statistical analysis

The data obtained in the study were tabulated in MS excel and the statistical analysis was done using SPSS software version 17.0 (IBM Corp., Armonk, NY and 2009). The mean values were compared between the right and left sides in both males and females using an unpaired t-test. And the mean values between males and females were compared using an independent t-test. The proximal, middle, and distal mean values were compared using one way-ANOVA test. The p-value <0.05 was considered statistically significant.

## Results

**Figure 1:**
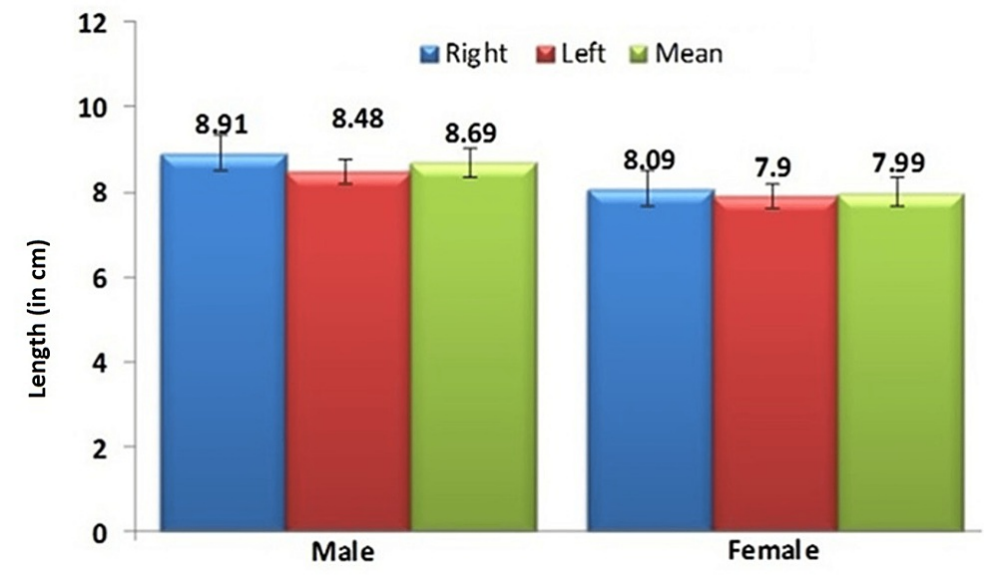
Comparison of Achilles tendon length in males and females

The average length of the AT was 8.69 ± 0.68cm in males and 7.99 ± 0.53cm in females, whereas the length of the right AT was 8.91 ± 0.7cm in males and, 8.09 ± 0.56cm in females and the left AT length was 8.48 ± 0.68cm in males and 7.90 ± 0.52cm in females were shown in Figure 1.

The length of AT was statistically higher in males than in females (p<0.001). The length was higher on the right side when compared with the left side in both genders and was showed statistically significant.

**Figure 2:**
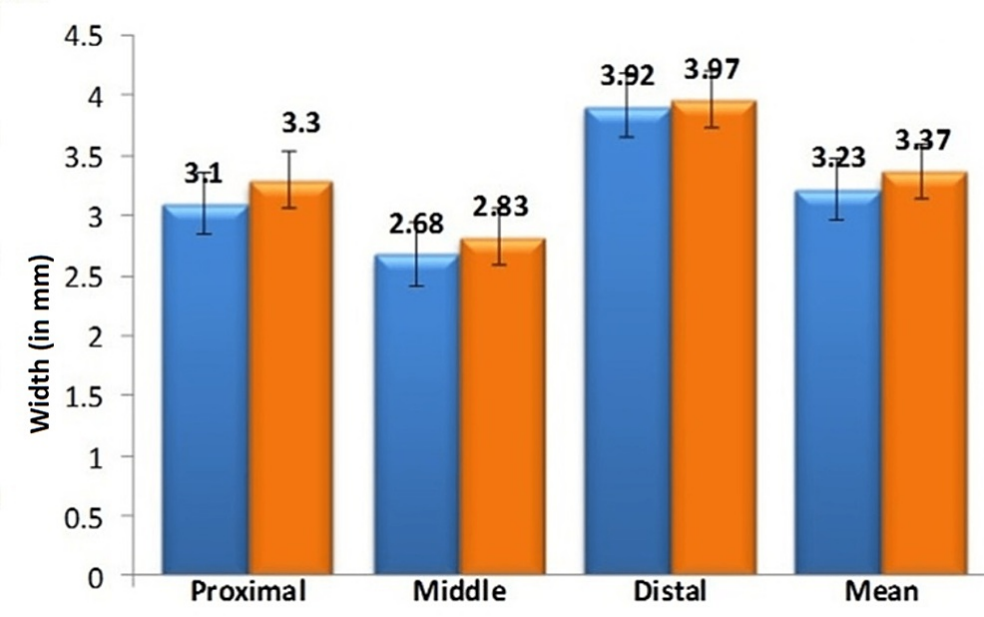
Comparison of mean Achilles tendon width in males at various levels

The width (in mm) of AT was measured at three different levels (proximal, middle, and distal). The mean width in males on the right side was PWAT - 3.1 ± 0.23mm, MWAT - 2.68 ± 0.26mm, DWAT - 3.92 ± 0.52mm, and on the left side was PWAT - 3.30 ± 0.29mm, MWAT - 2.83 ± 0.18mm, DWAT - 3.97 ± 0.40mm were shown in Figure 2.

**Figure 3:**
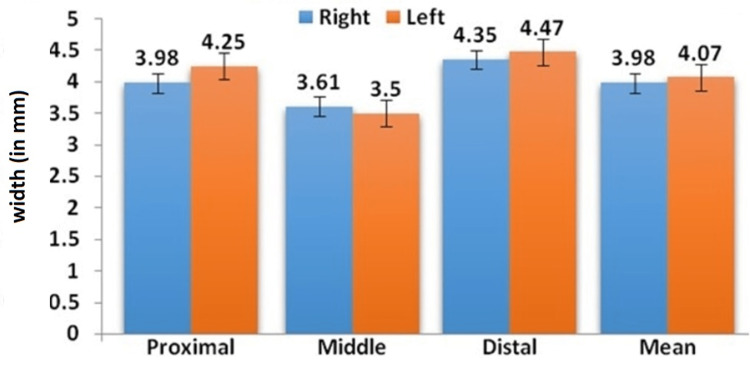
Comparison of mean Achilles tendon width in females at various levels

Similarly, the mean width in females on the right side was PWAT- 3.98 ± 0.38mm, MWAT-3.61 ± 0.16mm, DWAT 4.35 ± 0.53mm, and on the left side was PWAT - 4.25 ± 0.34mm, MWAT- 3.50 ± 0.2mm, DWAT- 4.47 ± 0.48mm were shown in Figure 3.

**Figure 4:**
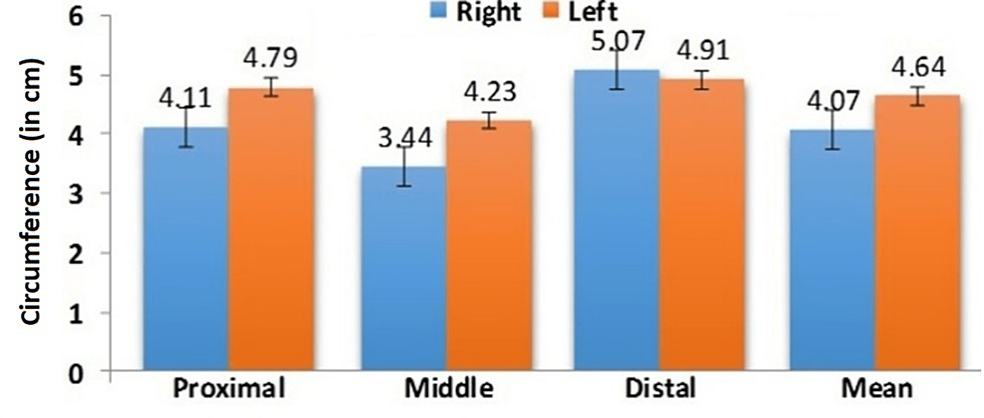
Comparison of mean Achilles tendon circumference in males at various levels

The width of AT in females was statistically higher than in males, similarly, the left side was significantly higher than the right side in both genders. The circumference of the AT varies in different regions, and it was analyzed in three different levels (proximal, middle, and distal). The circumference of the AT in males on the right side was PCAT - 4.11 ± 0.25cm, MCAT- 3.44 ± 0.14cm, DCAT - 5.07 ± 0.26cm, and on the left side, it was PCAT 4.79 ± 0.25cm, MCAT - 4.23 ± 0.14cm, DCAT- 4.91 ± 0.69cm were shown in Figure 4.

**Figure 5:**
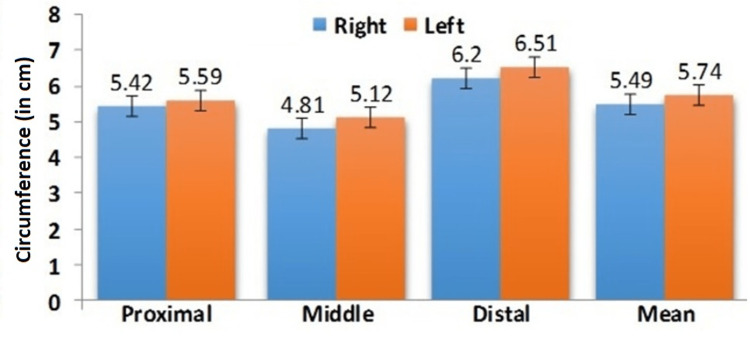
Comparison of mean Achilles tendon circumference in females at various levels

Similarly, in females on the right side was PCAT - 5.42 ± 0.37cm, MCAT 4.81 ± 0.26cm, DCAT - 6.20 ± 0.74cm, and on the left side was PCAT 5.59 ± 0.37cm, MCAT- 5.12 ± 0.43cm, DCAT - 6.51 ± 0.69cm were shown in Figure 5.

**Figure 6:**
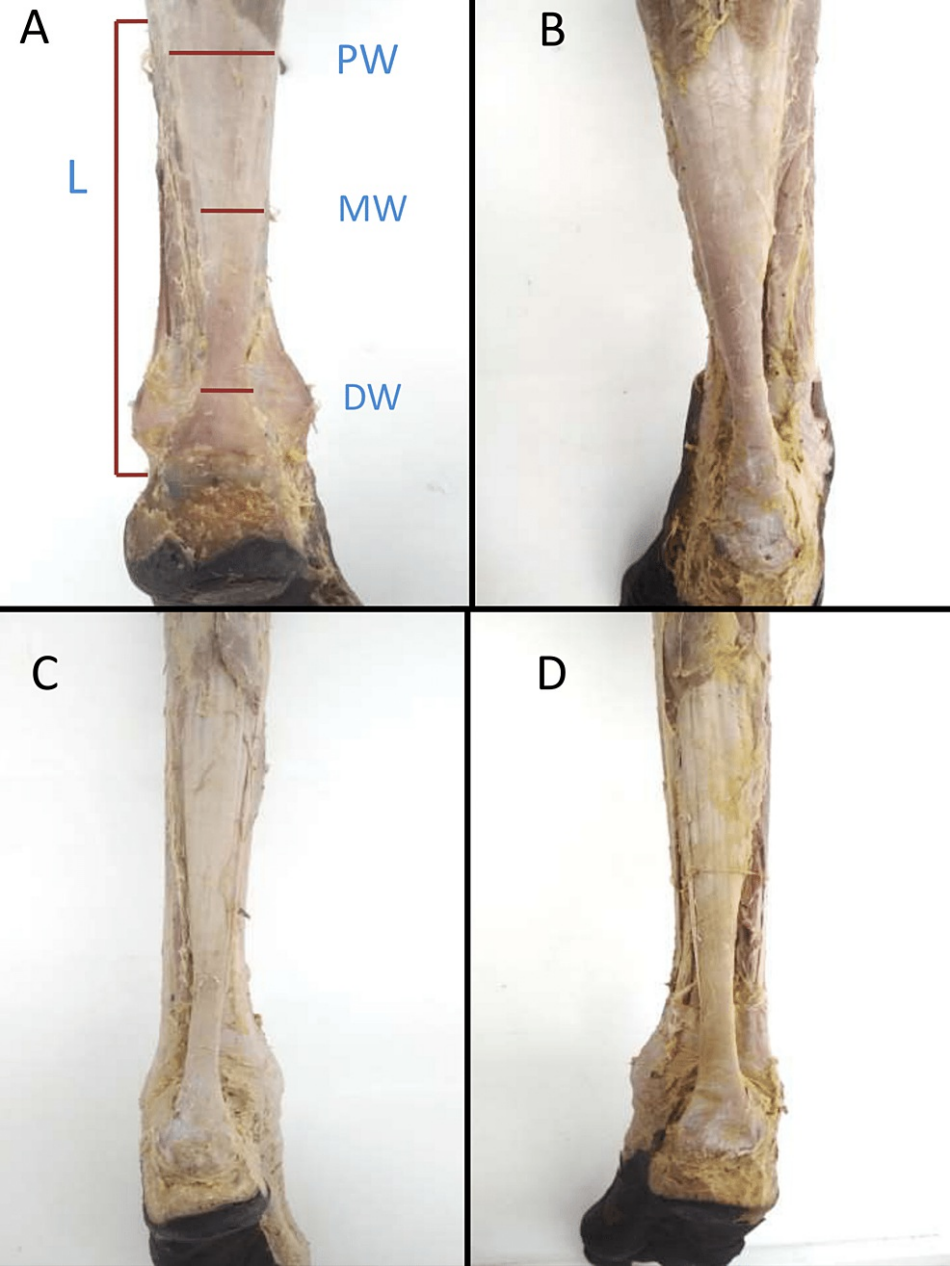
Morphometry of Achilles tendon in males and females (A) Achilles tendon male left leg, (B) Achilles tendon male right leg, (C) Achilles tendon female left leg, (D) Achilles tendon female right leg L - Length of Achilles Tendon, PW - Proximal Width, MW - Middle Width, DW - Distal Width.

The circumference of the AT in females was statistically higher than in males, similarly, the left side was significantly larger than the right side in both genders were shown in Figures 6A-6D.

Histology of AT

**Figure 7:**
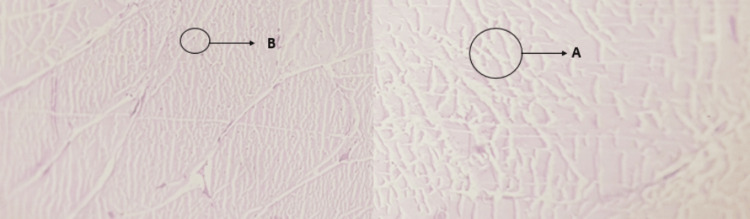
Histology of Achilles tendon using hematoxylin and eosin, magnification 10x A - Collagen Fibers, B - Fibroblast

Tendons have tightly packed collagen bundles arranged parallel in the intercellular ground substance. The AT of the male cadavers shown in Figure 7(A) have cells with oval nuclei parallel to the collagen fibers and fibroblast with minimalthe cytoplasm with same refractive index as the fibers. The female AT slide shows in Figure 7(B) cells with relatively small withinarranged within collagen fibers. The secretion of fibers collagen fiber by fibroblasts, arrests itself over time relatively in between the same fibres. These cells bea come small with stellate appearance and heterochromatare nuclei which are known as tendon cells, the responsible for restoration of injured tissue.

## Discussion

The estimated incidence of AT injury rate is 12 in 100,000 individuals which is more common in males than females with a ratio of 5:1 [16]. Acute or chronic AT injury is more frequent among the general population, especially in the geriatric age group and athletes due to disuse and overuse respectively, which results in discomfort and locomotor disability [2,17-19]. The males are prone to AT injury mainly due to higher body mass, strenuous activities, and increased muscle mass, and females are less prone due to a higher amount of estrogen levels, less strenuous activities, and decreased muscle mass [20]. Three vascular zones were observed over the skin of AT namely, medial (high vascularity), lateral (moderate vascularity) and posterior (poor vascularity) the zones are crucial, and surgeons should keep them in mind to make a line of incision and improve healing at the end of surgery [21].

In this research, the average length of AT in all 60 cadaveric lower extremity specimens was in both males 8.69 ± 0.68cm and females 7.99 ± 0.53cm. The present findings were closely correlated with Naveen et al. and Apaydin et al. showed that the average length of the AT is 10-15cm, ranging from 9 to 19cm [22,23]. The average length of right AT in males and females was 8.91 ± 0.7 and 8.09 ± 0.56cm, respectively, and the length of left AT in males and females was 8.48 ± 0.68cm and 7.90 ± 0.52cm. The length of AT showed no statistical differences between the right and left sides; on the contrary, there was a significant difference observed between male and female individuals. The results were similar to Naveen et al. [22]. and Canbolat et al. who showed no statistical difference in the length of right and left AT [24]. Modern humans, especially females, are more toward a sedentary lifestyle than males, the activities are reduced in females than in males and the length of the tenocytes will also reduce due to minimal stretching of fibers. The stiffness of the AT is more common in males than females due to maximal loading and lengthy AT, because of which various deficits are more common, among males than females especially tendinopathy, etc. Contrary to this, another study showed that females have higher CSA deformation under maximal voluntary isometric contraction (MVIC), indicating that a more transversely compliant tendon is present compared to males. Thus, these findings confirm the hypothesis that females will demonstrate higher AT-CSA deformation and compliance during contractions compared to males [25]. In order to avoid these complications, the individual has to walk regularly, and the athlete individual has to undergo proper maintenance of AT with the correct frequency, duration, and intensity exercise regimen, to prevent degeneration and stiffness. The present length of AT is compared with the previous data, and they are expressed in Table 1.

**Table 1:** Comparison of Achilles tendon length with previous literature

Authors & Year	Type of study	No of lower limb used	Mean length of right Achilles tendon in cm (Mean ± SD )		Mean length of left Achilles tendon in cm (Mean ± SD )	
			Male	Female	Male	Female
Fukutani et al. 2014 [26]	Ultrasonography	10	5.6 ± 4.6 mm		9.0 ± 5.4 mm	
Pang et al. 2006 [27]	Ultrasonography	40	11.74 ± 24.71		11.72 ± 22.87	
Chhiring et al. 2022 [28]	Cadaver	40	16.65 ± 1.72 cm	-	16.35 ± 1.49 cm	-
Szaro and Ghali 2021 [29]	MRI	74	8.1cm	-	-	-
Manju et al. 2019 [30]	Cadaver	108	7.24 ± 2.57		6.34 ±1.61	
Nahar et al. 2019 [31]	Cadaver	60	14.74 ± 2.25	14.62 ± 2.46	12.77 ± 2.11	12.56 ± 2.21
Kumar et al. 2017 [22]	Cadaver	64	7.8 ± 1.9	-	7.5 ± 2.2	-
Patel and Labib 2017 [32]	Ultrasonography	50	9.32 ± 1.67		9.39 ± 1.59	
Wren et al. 2010 [33]	Cerebral palsy children	40	0.47		-	-
Zellers et al. 2018 [34]	Ultrasonography	42	20.7			
Present study	Cadaver	60	8.91 ± 0.56 8.09 ± 0.7		8.48 ± 0.52 7.90 ± 0.68	

The findings revealed that the circumference of AT varies at three different levels. The circumference of the distal segment is higher, followed by the proximal and middle regions. In comparison within genders, the circumference was significantly higher in females than males due to more sedentary lifestyles and hormonal responses. Similarly, the findings of this study were correlated with the Lama et. al., conducted in the cadaver [28]. On comparing the sides there was a statistically significant difference between the right and left sides. The left side circumference was higher than the right side at various levels and the difference was observed between the genders. The midportion of the AT is thinner mainly due to the presence of minimal vascularity and connective tissue structures than the insertional and non-insertional regions of AT. This difference in vascularity results in tremendous transmission of mechanical loading and hence is the more common site for injury, especially in tendinopathy than the other two sites. The shear wave velocity decreases in thin AT and increases with thick AT. In addition, the velocity of the shear wave increased during dorsiflexion than plantar flexion [35]. The elastic properties of the AT reform in geriatric population than young individuals and in addition the thickness of AT declines resulting in an increase in stiffness [36,37]. In the comparison of the general population with athletes, athletes are continuously involved in various sports activities and their tendons are more common for injury than the geriatric and general population. The circumference of AT plays a major role in the formation of tendinopathy, rupture, degenerative changes, and other related diseases. In addition to that thin AT is commonly associated with pes cavus and could result in various secondary complications of the low back region, hip, knee, and foot [38]. The Achilles tendinopathy was considered only if the circumference exceeds 8mm or more [39]. Hypovasularity in the midportion is one of the significant factors for delayed healing in proximal and middle regions [12,19]. The knowledge regarding the circumference of AT is crucial for surgeons while performing surgeries in AT and in addition the AT is also used as an allograft for cruciate ligament injury in athletes [40]. The circumference of AT observed in the present study was compared with the previous data and they were expressed in Table 2.

**Table 2:** Comparison of Achilles tendon width with previous literature

Authors & Year	Type of study	Total no limbs	Mean width of proximal segment of right and left Achilles tendon in cm (Mean ± SD )				Mean width of mid-region of right and left Achilles tendon in cm (Mean ± SD )				Mean width of distal segment right and left Achilles tendon in cm (Mean ± SD )			
			Male		Female		Male		Female		Male		Female	
			R	L	R	L	R	L	R	L	R	L	R	L
Chhiring Palmu Lama, 2022 [28]	Cadaver	40	5.50 ± 1.07	5.25 ± 0.88	-	-	-	-	-	-	2.22 ± 0.54	2.05 ± 0.27	-	-
Manju Singhal, 2019 [30]	Cadaver	108	1.45 ± 0.18	1.35 ± 0.25	1.23 ± 0.33	1.40 ± 0.22	1.23 ± 0.30	1.25 ± 0.28	1.25 ± 0.45	1.23 ± 0.45	2.33 ± 0.52	2.25 ± 0.46	2.13 ± 0.63	2.18 ± 0.62
Nahar L 2019 [31]	Cadaver	60	5.87 ± 0.84	5.80±0.86	4.49±0.55	4.33±0.57	1.65±0.44	1.51±0.44	1.48±0.36	1.41±0.37	2.84±0.52	2.71±0.44	2.44±0.45	2.37±0.45
Nick N. 2017 [32]	Ultrasonography	50	-	-	-	-	-	-	-	-	1.38 ± 0.16	1.34 ± 0.13	1.52 ± 0.14	1.40 ± 0.16
Naveen Kumar 2017 [22]	Cadaver	64	2.2 ± 0.4	2.0 ± 0.4	-	-	-	-	-	-	2.4 ± 0.3	2.5 ± 0.5	-	-
Pallavi Bajpayee [41]	Cadaver	70	-	-	-	-	1.49 ± 0.24	-	-	-	3.5 ± 0.54	-	-	-
Present study	Cadaver													

The findings of this research revealed that the width of the AT differs among male and female individuals. In addition, there is also a notable difference observed between the right and left sides of an individual, but it is not statistically significant. In this study, three different levels of AT width were assessed, and it was compared with the right and left sides and between the sex of the individuals. The present study showed that the mean width of the AT is higher in the distal segment (point of insertion), followed by the proximal and midportion of the AT. The present findings were correlated with the lama et al, conducted research in cadavers with only males [28]. On the contrary, Abdel-Ghany and Ollo and Chen e al. found that the breadth of the male tendon Achilles was higher at the distal region and it was higher on the right side than the left side [42,43]. In the case of females, no statistical difference was seen between the right and left sides. Chen et. al. reported that the breadth of the tendocalcaneum was 2.2 ± 0.29cm at the level of its junction with calcaneus [43]. The values were quite smaller than the present study and the difference may be due to differences in race, ethnicity, and BMI.

The extensive length of the AT in humans is crucial for athletic and non-athletic individuals to perform efficient activities like walking, jumping, and running through stretching and by the breakdown of ATP [44,45]. Children with normal day-to-day activities will develop extensive AT than children having delayed milestones in certain conditions like cerebral palsy, congenital talipes equinovarus, etc. During extended stress, the collagen bundles of the tendon are arranged in a specific pattern. In response to prolonged stresses applied in the same direction, this tissue's collagen fibers align with the fibroblast's linear orientation, providing excellent resistance to traction forces [46,47]. Dense connective tissue has evolved to provide resistance and protection. There are a variety of molecular components present in different regions of the AT to withstand mechanical loads. The proximal region has less amount of fibro cartilaginous tissue with a minimal amount of aggrecan and biglycan to withstand the tensile forces, while the distal region has the maximal amount of fibro cartilaginous tissue to withstand the compressive forces. It is mainly made up of type I collagen (90%) and during the healing process after the injury, it modifies to type III collagen fibers. In addition to fibers, it has a minimal amount of proteoglycans (PGs) namely decorin and fibromodulin which helps in anchoring the fibrils in the AT [48,49]. During various day-to-day activities in the individual life, the amount of load transmission in the AT tendon varies. During activities like walking, hopping, squat jumping, jumping, and running the AT yields a load of 1.9-9 kN respectively [1,50,51]. 

Limitations of the study

The present study has been done in cadavers with minimal sample size and the correlation between the length and anthropometry was also not assessed. The increased sample size and live human study with UTE MRI, and ultrasound could give precise findings and could help in identifying the reliable risk factors from tendinopathy to injury. In addition, complex imaging techniques like sonoelastography and intra-molecular composition and fibers will give ideal information on the AT.

## Conclusions

This study confirms that AT length was significantly higher in males than females, whereas on comparing width and circumference were higher in females than in males. In addition, the left-side parameters were showed significant than the right side. The knowledge regarding the morphometry of AT can inculcate novel approaches in the repair, reconstruction, and diagnosis of AT deficits by various medical professionals like orthopedic surgeons, podiatrists, sports physicians, and physiotherapists.
